# Retraction Note: Topical antibiotics reduce CD11c+ cell numbers in the healthy murine cornea and modulate their response to contact lens wear

**DOI:** 10.1038/s41598-025-00282-1

**Published:** 2025-05-12

**Authors:** Ananya Datta, Justin Lee, Tiffany Truong, David J. Evans, Suzanne M. J. Fleiszig

**Affiliations:** 1 Herbert Wertheim School of Optometry & Vision Science, University of California, Berkeley, CA 94720 USA; 2 College of Pharmacy, Touro University California, Vallejo, CA USA; 3 Graduate Groups in Vision Science, Microbiology, and Infectious Diseases and Immunity, University of California, Berkeley, CA USA

Retraction of: *Scientific Reports* 10.1038/s41598-022-14847-x, published online 23 June 2022

The Authors have retracted this Article. Following detection of duplicated images in two panels, the Authors carried out a broad investigation of the raw data contributing to all 10 panels in Figs. 2 and 3. The raw data consisted of confocal image Z-stacks through the entire thickness of the cornea of mouse eyes, each composed of many images. These were used to reconstruct corneal tissue regions in 3D, before the number of cells present was quantified using Imaris software (quantitative results shown in Figs. 2b and 3b). To produce the panels in Figs. 2a and 3a, Imaris software was used to collapse the image stacks, thereby allowing all cells in representative 3D tissue blocks to be viewed in 2D. Thus, the images shown in the panels were not individual captured images, and the cells shown in each were not necessarily all in the same plane. After reviewing the original files stored on the confocal microscope used to capture the images, the Authors found unexplained inconsistencies in file labeling that have undermined their confidence in the conclusions of the paper.

Ananya Datta and Suzanne M. J. Fleiszig agree to retract this article. Justin Lee, Tiffany Truong and David J. Evans did not respond to correspondence from the publisher regarding the retraction.

